# The effects of *Crataegus pinnatifida* (Chinese hawthorn) on metabolic syndrome: A review

**DOI:** 10.22038/IJBMS.2019.31964.7678

**Published:** 2019-05

**Authors:** Shahrzad Dehghani, Soghra Mehri, Hossein Hosseinzadeh

**Affiliations:** 1 Department of Pharmacodynamy and Toxicology, School of Pharmacy, Mashhad University of Medical Sciences, Mashhad, Iran; 2 Pharmaceutical Research Center, Pharmaceutical Technology Institute, Mashhad University of Medical Sciences, Mashhad, Iran

**Keywords:** Crataegus pinnatifida, Diabetes, Dyslipidemia, Hawthorn, Metabolic syndrome, Obesity

## Abstract

Metabolic syndrome is described as a group of risk factors in which at least three unhealthy medical conditions, including obesity, high blood sugar, hypertension or dyslipidemia occur simultaneously in a patient. These conditions raise the risk for diabetes mellitus and cardiovascular diseases. Many recent studies have focused on herbal remedies and their pharmacological effects on metabolic syndrome. *Crataegus pinnatifida* or Chinese hawthorn has been widely used in the treatment of hyperlipidemia and cardiovascular diseases. Its leaves, fruits and seeds have various active substances such as, flavonoids, triterpenic acids and sesquiterpenes, which through different mechanisms can be beneficial in metabolic syndrome. Flavonoids found in the leaves of hawthorn can significantly reduce atherosclerotic lesion areas, the fruit extracts contain two triterpenic acids (oleanolic acid and ursolic acid), that have the ability to inhibit the acyl-coA-cholesterol acyltransferase (ACAT) enzyme and as a result reduce very low-density lipoprotein (VLDL) and low-density lipoprotein (LDL) cholesterol levels. Another example regards a sesquiterpene found in the seeds of *C. pinnatifida*, which exhibits the ability to inhibit platelet aggregation, thus showing antithrombotic activity. Various studies have shown that *C. pinnatifida *can have beneficial effects on controlling and treating high blood sugar, dyslipidemia, obesity and atherosclerosis. The aim of this review is to highlight the interesting effects of *C. pinnatifida *on metabolic syndrome.

## Introduction

Metabolic syndrome is an important cause of mortality in both developing and developed countries (1). Metabolic syndrome is a condition in which many different combinations of unhealthy circumstances including hypertension, dyslipidemia, obesity, insulin resistance, and type 2 diabetes occur in a patient. These diseases create major health problems worldwide (2). The exact pathology of metabolic syndrome is still unknown, but studies have found that abnormal lipid levels could be a major contributing factor and a distinct feature of this disease (3). One underlying condition involved in the formation of metabolic syndrome, hyperlipidemia, is also known to be a major factor in the development of cardiovascular diseases. Another contributing factor, obesity, is also a risk factor for diabetes and cardiovascular diseases (4). Diabetes itself is quickly becoming a very common disease worldwide. In Asia alone, diabetes is expected to rise 2-3 folds by 2030 (5). Apart from the major cardiovascular problems of diabetes, conditions like polyuria, polyphagia, and polydipsia are also associated with this disease (6). Fatty liver disease is also associated with obesity and metabolic disorders. Studies have shown that an increase in dietary saturated fat can lead to its accumulation in different organs such as the liver (7).

There are certain medications for the treatment of these diseases. However, the majority of these medications are often associated with adverse reactions and side effects. Thus, researchers are always searching for better, safer and more natural replacements. For thousands of years, herbal medicines have been used to treat diseases in Eastern countries. In the last twenty years, these drugs have become more popular in the west, as well (8, 9). Studies have shown that herbal remedies have protective effects against chemical and natural toxins. To name a few examples, experiments have shown that saffron displays protective effects against organ toxicities caused by anti-tumor drugs and can protect various organs and tissues from both natural and chemical toxins. Furthermore, saffron and its components display antidotal properties for natural toxins such as, mycotoxins and endotoxins (10). Other examples are cinnamon and green tea, which are both herbal remedies as well; they also show protective properties against various toxins (11, 12). A great deal (more than 80%) of the population in developing countries uses traditional herbal medicines to treat diseases (13). Several plants have been discovered to be useful in metabolic syndrome, for instance avocado (14), saffron (15), cinnamon (16), red pepper (17), garlic (18), and grapes (19). The Chinese Drug and Food Administration have approved fifty-seven traditional Chinese medicines (TCM) for the treatment of hyperlipidemia. Among these TCMs, hawthorn fruit (*Cratageus pinnatifida*) or Shan zha is prescribed in more than 50% of the formulations, making it the most popular TCM to treat hyperlipidemia (20).

**Figure 1:**
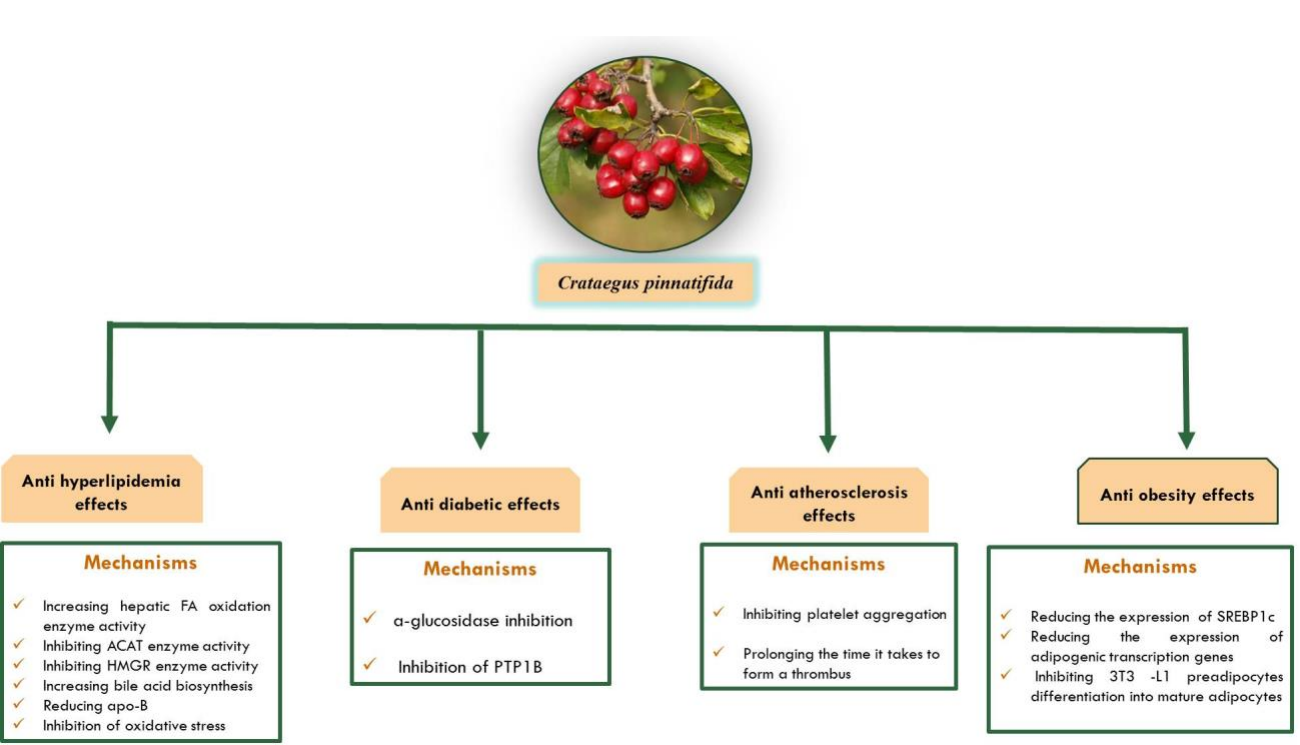
Metabolic effects and mechanisms of action of *Crataegus pinnatifida*

Hawthorn belongs to the *Crataegus *species and *Rosacea *family. It has been used as a fruit in China dating back to the seventh millennium before Christ (21). According to the British Herbal Pharmacopoeia, (1983) Hawthorn (*C. pinnatifida*) has been used to treat myocardial dysfunction, hypertension and atherosclerosis. It has also been used to treat blood circulation problems in China (22). Studies have shown that the extracts prepared from the fruits, leaves and flowers of *C. pinnatifida *may be able to prevent heart failure and hypertension (23). One of the most important effects of hawthorn is its ability to reduce serum cholesterol (according to animal studies) (24) and to have anti-diabetic effects. Regarding to different pharmacological effects of *C. pinnatifida*, our aim in this paper is to review the main effects of this plant on metabolic disorders. For this purpose, we searched PubMed and Science Direct databases for keywords containing metabolic syndrome, *C. pinnatifida*. Hawthorn, hyperlipidemia and others until the end of October 2017. Figure 1 shows the metabolic effects of C*. pinnatifida*.

**Figure 2:**
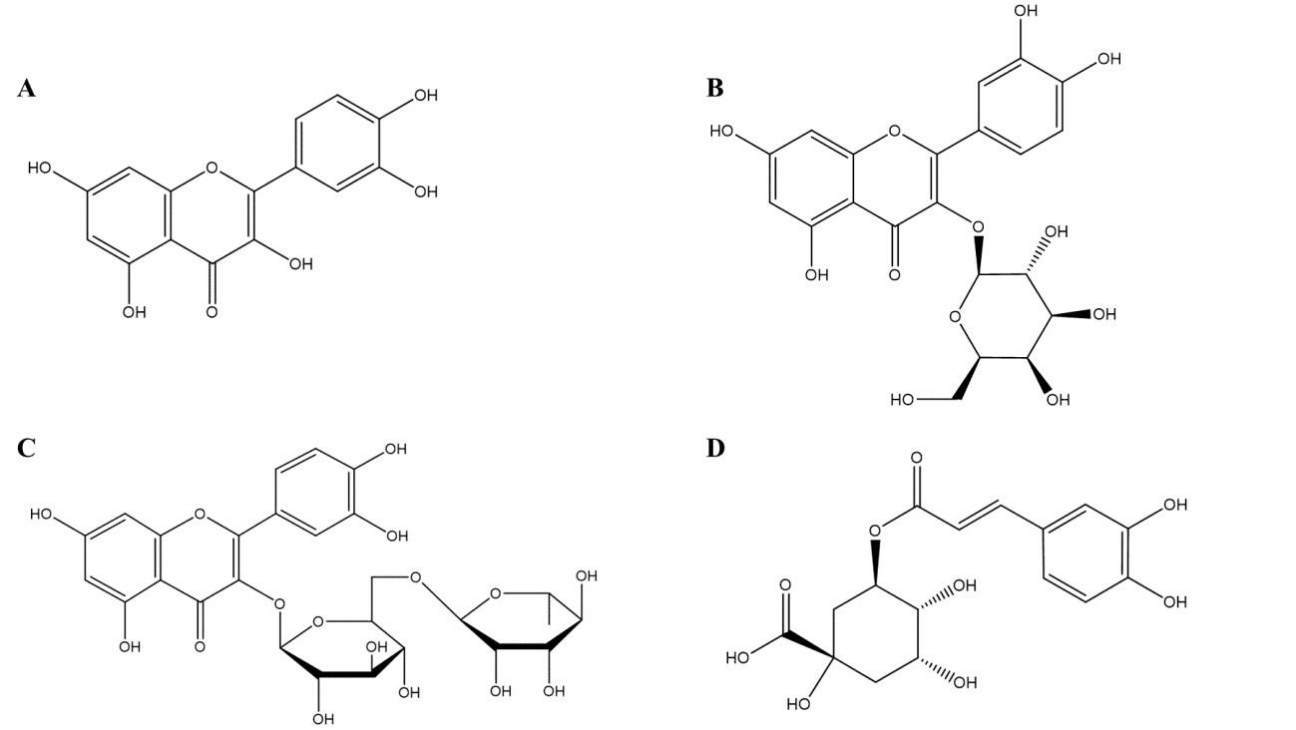
The chemical structures of the different constituents of the hawthorn fruit with HMGR inhibitory effects

**Figure 3:**
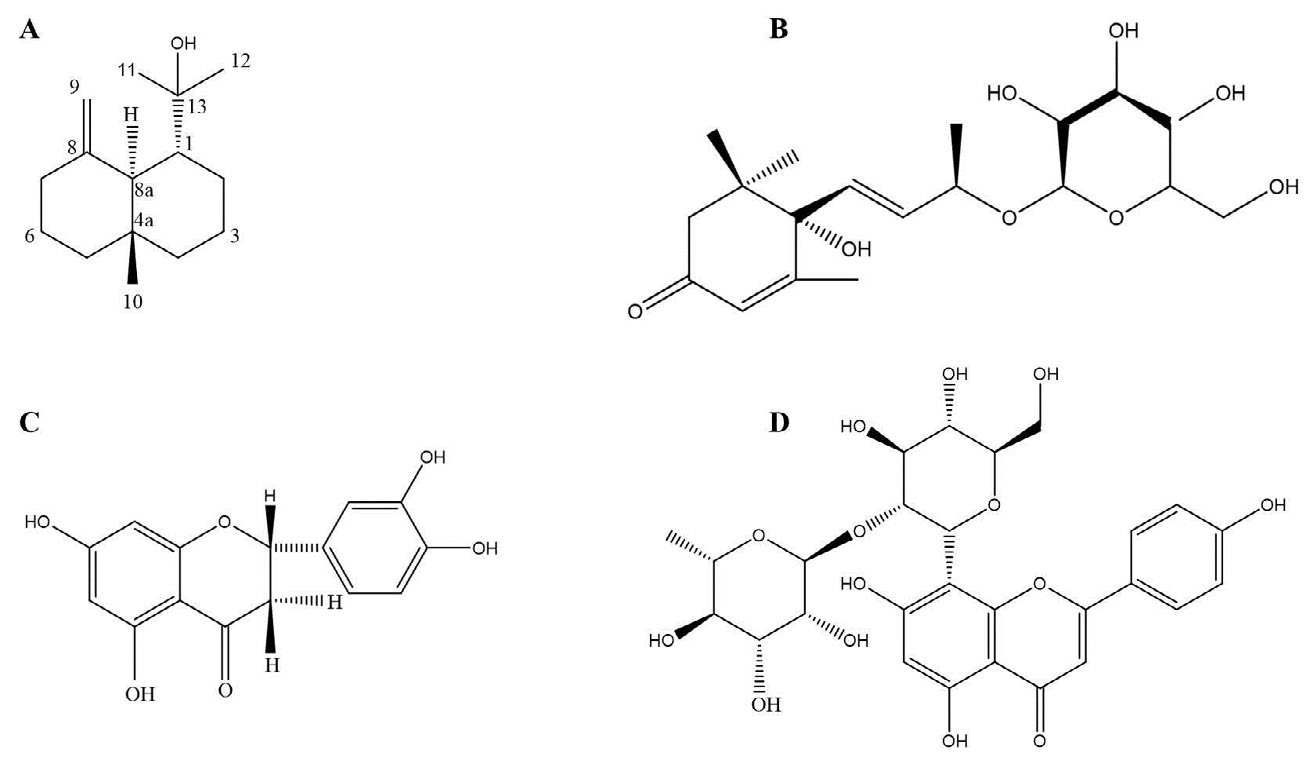
The chemical structures of the different constituents of the hawthorn fruit with antithrombotic activities

**Table 1:** Different studies on anti –hyperlipidemia effects of *Crataegus pinnatifida*

**Part(s) of the plant used/Extract(s)**	**Active** **constituents**	**Dosage/route/duration of treatment**	**Experiment model**	**Outcome**	**Reference**
Fruit	Pectin penta - oligogalacturonide	300 mg/KG concentration/oral infusion/ 4 and 10 weeks	Six week old male Kunming mice	Hepatic TG was suppressed by as much as forty percent. HPSS administration was also able to reduce lipid inclusions in the liver by number and size as well. In addition, epididymal and perirenal fat accumulation was suppresed.	(50)
Fruit extract	Oleanolic acid (OA) and ursolic acid (UA) (two triterpenic acids)	Different dosages/oral/4 weeks	Male Lakeview golden Syrian hamsters	By inhibiting the ACAT enzyme, OA and UA reduced LDL and VLDL cholesterol levels by 6% compared to the control.	(51)
Fruit extract	3-hydroxy-3-methylglutaryl coenzyme A reductase (HMGR) inhibitors	Stomach tube/six weeks	Kunming mice	Results identified 4 main HMGR inhibitor compounds, Quercetin, Hyperoside, Rutin and Chlorogenic acid.	(53)
A multi herb preparation Multiherb formula (*Crataegus pinnatifida, Alisma orientalis, Stigma maydis, Ganoderma lucidum, Polygonum multiflorum and Morus alba*)		Four capsules of either the placebo or herbal formula in the morning and in the evening/oral 12 weeks	42 Chinese patients 18 years or older considered to have dyslipidemia	Results showed that there was a 9.4 % decrease of LDL- C; the study also showed that after 12 weeks, the herbal formula elevated triglyceride levels.	(54)
Multiherbal preparation containing *Aurricularia aurricula, Pueraria* *radix* and *Crataegus pinnatifida* (AHP)	Polysaccharides and polyphenols	150mg/kg/day and 450 mg/kg/day/ gastric infusion/ 12 weeks	4 week old ICR mice	At the dose of 150mg/kg/day, AHP reduced LDL-C by 22.92 % and at the dose of 450 mg/kg/day LDL-C decreased by 39.58%. Total cholesterol levels were also reduced to 75.13% (150mg/kg/day) and 68.81 %( 450 mg/kg/day) of the initial levels in the CED group. AHP also showed the ability to decrease fatty deposits in the liver of the treated mice.	(55)
Extract from fruit powder	-	Either 100mg/kg of the hawthorn extracts(OL) or 200mg/kg of the extract(OH)/oral/8 weeks	Nine week old female Sprague –Dawley rats (ovariectomized or sham group)	Total cholesterol levels were reduced by 16% in the OL group and 14% lower in the OH group compared to the OVX control group, which did not receive hawthorn fruit extract. Also LDL levels decreased by 20 and 32 % in the OL and OH groups respectively. Free fatty acids decreased by 51% in the OH group compared to the OVX control. However no significant difference was observed in triglyceride and VLDL levels	(57)
Shan-Zha (fruit of *Crataegus pinnatifida*)	-	250mg/kg Shan-Zha (which was dissolved in water) was administered by gavage orally 3 times a day for a week.	C57BL/6J male mice	Results showed that treatment with Shan-Zha was able to lower both triglyceride and cholesterol levels. However, it did not reduce body weight in the HFD mice.	(60)
Dried powder of hawthorn fruit	-	2% w/w powder fed for 4 weeks	Male Sprague Dawley rats	Total cholesterol and LDL levels were reduced. An increase of HDL levels was observed.	(62)
A low sugar content drink from hawthorn. It was also fortified with vitamin c and zinc gluconate to enhance the anti-oxidative properties of the plant	-	Fed the Hawthorn drink for 6-9 weeks	37 male Sprague-Dawley rats	After the tenth week of treatment, the group receiving the hawthorn drink showed significantly lower body weights. The serum cholesterol and TG levels were also reduced significantly. Results also showed that the hawthorn drink was able to increase HDL-C levels and decrease LDL-C levels the rats.	(63)
A low sugar content drink from hawthorn. It was also fortified with vitamin c and zinc gluconate to enhance the anti-oxidative properties of the plant	-	hawthorn drink twice a day for a month	Humans that were diagnosed with hyperlipidemia	The patients had an initial average serum cholesterol level of 7.30 ± 1.04 mmol/l which was reduced to 6.19 ± 1.56 mmol/l after one month of drinking the hawthorn drink. Their triglyceride levels also decreased from 1.93 ± 0.92 to 1.75 ± 0.96 mmol/l. The drink was also able to lower LDL-C levels.	(63)

**Anti-diabetic effects**

Diabetes is a worldwide known disease in which there is an abnormal increase in blood glucose levels. Data show that in 2015, 415 million adults between the ages of 20 and 70 were diagnosed with diabetes. This number is expected to rise to 642 million by the year 2040 (25).

Control of postprandial hyperglycemia in order to avoid sharp blood glucose peaks has become an important basis for new medicinal approaches in the treatment of diabetes (for instance by using drugs such as acarbose, miglitol and voglibose) (26). In a study performed in 2011 (27), the effects of *C. pinnatifida *leaf extracts were demonstrated to be helpful in lowering postprandial hyperglycemia in male Wister rats. In this study, the sample prepared by extracting the hawthorn leaves with 70 percent ethanol, was able to inhibit plasma glucose increase at doses of 500 mg/kg after 30 min administration of sucrose. Previous studies have suggested that *C. pinnatifida *leaf flavonoids have inhibitory effects on rat intestinal enzymes such as, α-glucosidase. Thus it may be assumed that the leaf flavonoid fraction lowers plasma glucose through this mechanism (28).

Improving the gastrointestinal transit is also believed to be beneficial in the treatment of postprandial hypertriglyceridemia in diabetic patients, possibly, by decreasing the rate of gluconeogenesis (29). In order to prove this hypothesis, once again, the sample was prepared by extracting the hawthorn leaf with ethanol and the animals used were male ddY mice. The samples of hawthorn (especially at the dose of 500 mg/kg) were able to show accelerating effects on gastrointestinal transit (27).

In a recent study, focusing on the anti-diabetic effects of the fruit of *C. pinnatifida, *the fruits were dried, ground to powder, and then extracts were prepared with different solvents (for example ethyl acetate (EtOAc) and methanol (MeOH)). The extracts were shown to have *in vitro* α-glucosidase inhibitory potential. The results showed that the EtOAc fraction had the most active α-glucosidase inhibitory effect, with an IC_50_ of 22.70 µg/ml compared to acarbose with the IC_50_ of 81.65 µg/ml (positive control). The MeOH extract and some of the other fractions of *C. pinnatifida *were shown to have a-glucosidase inhibitory effects, as well. Experiments through column chromatography were shown that the different extract factions contain active compounds such as, hyperoside, chlorgenic acid and tripenic acids (ursolic acid, oleanolic acid and 3-epicorosolic acid). Chlorgenic acid and hyperoside were isolated from the EtOAc fraction and have already been found to have α-glucosidase inhibitory effects. Ursolic acid and oleanolic acid were isolated from the CH_2_Cl_2_ fraction and have been reported to be potent α-glucosidase inhibitors. All the compounds mentioned have also been proven to have protein-tyrosine phosphatase 1B (PTP1B) inhibitory effects. PTP1B is an enzyme that plays a key role in insulin regulation. 3-Epicorosolic acid (also isolated from the CH_2_Cl_2_ fraction), showed to be an uncompetitive inhibitor against α-glucosidase and a mixed type inhibitor of PTP1B (30).

Insulin resistance is a common underlying factor in the formation of several diseases like obesity, dyslipidemia, hypertension, type 2 diabetes, and coronary diseases (31). Studies show that insulin resistance can be a contributing factor for the development of non-alcoholic fatty liver disease (NAFLD) (32) which consists of a spectrum of problems starting from the deposition of triglyceride (TG) in the liver and then steatohepatitis and finally to fibrosis and cirrhosis (33).

Although there are different drugs that are useful for the treatment of these diseases, they also result in several adverse effects (34). Thus, naturally we turn to medicinal herbs to possibly find a new pathway in resolving these diseases in a safer way (35). It has been shown that not only using one herb but also combining them can prove to be useful for anti-diabetic treatments. For example, the combination of four herbs; *Puerariae radix*, *Lycium babbarum*, *Polygonati rhizome*, and *C. pinnatifida *(PLCP) were studied on insulin resistance and hepatic steatosis. Mice treated with ethanol or aqueous extracts of these plants showed sustained hypoglycemic effects. During the ninth week the area under the curve for EEM (medium dose of ethanol extract) and EEH (high dose of ethanol extract) decreased by 14.4 and 23.9 percent, respectively. EEH even performed better than the metformin group (MET) by preventing high glucose levels at 30, 60 and 120 min compared to the metformin group that only proved to be useful at 120 min. Insulin resistance (IR) was measured by HOMA-IR (Homeostatic Model Assessment of Insulin Resistance) and the results showed that although IR indices did improve with all three, EEM, EEH, MET groups, metformin was more capable in lowering insulin resistance, whereas the other two were more effective in reducing glucose levels in the fasting state. Although we should also include that the amelioration of IR with EEH and EEM reached similar levels of metformin after ten weeks of treatment. (36). By studying the photomicrographs of the liver histology for each group of mice, the model control group showed severe NAFLD (high lipid accumulation in the cytoplasm of liver cells). Results also showed the high accumulation of fat in hepatocytes in the metformin group. However, the EEM, EEH, and AEM (medium dose aqueous extract) groups both showed alleviation in hepatic steatosis (reduced surface area of steatosis) (36).

In a very recent 2017 report, the hypoglycemic effects of hawthorn in type 2 diabetes mellitus rat models was studied. Fifty-four male Sprague-Dawley rats were randomly divided into different groups, the normal control group, the T2DM (type 2 diabetes mellitus) induced group, and the other groups which received different doses of hawthorn fruit extract 50, 100 and 200 mg/kg (intra-gastric administration twice a day for two weeks). Fasting blood glucose levels were determined after two weeks of treatment with hawthorn extract and results showed that compared to the control groups hawthorn significantly reduced the glucose levels in the rat models at all three doses mentioned. Serum insulin levels significantly increased in the hawthorn treated group compared to the T2DM group. It has been speculated that polyphenol compounds in the hawthorn extracts may have useful hypoglycemic effects in the treatment of type 2 diabetes. The study did not determine the specific active compounds responsible for the observed effects and did not mention the exact mechanisms by which insulin levels were raised (37).

Overall studies performed show that *C. pinnatifida *has a great potential for the treatment of diabetes. Although different mechanisms and pathways have been studied in order to explain these results, more studies should be done to further pinpoint the exact mechanisms by which this herb performs its therapeutic effects.

**Anti-obesity effect**

Obesity is a serious disease that has been developing over the years and has turned into a global problem. Excess body weight is an important risk factor (sixth factor) in causing worldwide diseases. Obesity brings down life expectancy and unfortunately is seen in one billion adults in our population and also 10 percent of children (38). Obesity results due to an imbalance between the amount of energy we consume and the amount of energy we use, or burn (39). The current drugs used to treat obesity show to have side effects like GI disturbances, insomnia, headaches, diarrhea and flatulence (40, 41). Thus, finding a safer natural remedy could prove to be useful for patients worldwide.

In a recent 2015 study, the anti-obesity effects of a combination of *C. pinnatifida* leaf and *Citrus unshia* peel extracts (HTO48) were explored. *In-vivo* studies were performed on male Sprague Dawley rats. The rats were divided into two groups of chow diet group –and high fat diet group (HFD). Results showed that 0.2% and 0.6% concentrations of HTO48 (for 12 weeks) were able to reduce serum total cholesterol (TC) by 14.4% and 16.3 % and triglyceride (TG) serum levels by 33.7 % and 31.9% in HFD-fed rats compared to the HFD control group. It was also shown that HTO48 was able to significantly reduce the expression of sterol regulatory element-binding protein 1c (SREBP1c) and fatty acid synthase (FAS) genes, which are lipogenic genes that were higher in the HFD groups compared to the rats receiving a normal chow diet. Adipogenesis is a process in which preadipocytes mature into adipocytes by the enlargement of intracellular lipid droplets (a process involved in the formation of obesity) (42). In the study, the effects of different doses of HTO48 were examined on 3T3 -L1 preadipocytes by observing the expression of genes related to adipogenesis (PPAR Gama, aP2 and LPL mRNA). It was shown that HTO48 was able to significantly reduce lipid droplet formation and TG accumulation, thus inhibiting 3T3 -L1 preadipocytes differentiation into mature adipocytes. HTO48 was also able to reduce the expression of adipogenic transcription genes [peroxisome proliferator-activated receptor alpha (PPARg) and C/EBPa mRNA] and stimulate glycerol release (promoting lipolysis) in 3T3 -L1 adipocytes. Other results of the study also suggest that HTO48 can control weight gain and serum lipid levels, by inhibiting adipose tissue accumulation and increasing excretion of lipids through feces. However, these effects only were evaluated on animal models fed a high fat diet and did not consider the effects of HTO48 on chow diet taking models. It should also be noted that, specific active compounds were not extracted for further evaluations. To conclude the results suggested that with further experimental assays, HTO48 could be considered as a fine candidate for treating obesity and hyperlipidemia in the future (43).

*C. pinnatifida* (Shan zha) fruit extracts were used in a study to see if they had an effect on obesity and dyslipidemia in hamsters receiving a high fat diet. For this study 40 male Syrian golden hamsters weighing 130-140 g were fed a high fat diet for 7 days, then they were divided into different groups each receiving different test compounds (like; Clofibrate, 250 mg/kg of Shan zha and others) while one group was considered the control group (received the vehicle only). Results showed that the group, which received 250 mg/kg of Shan zha (dissolved in water), had a 15% lower plasma total cholesterol level and 20% decrease of low-density lipoprotein cholesterol (LDL- C) after 7 days of treatment. While high-density lipoprotein cholesterol (HDL-C) levels were elevated by 27% after 7 days of treatment. Results also showed that hamsters that were fed the Shan zha had significantly lower body weights and decreased adipose tissue, compared to the control group. Through Western blot analysis, it was speculated that Shan zha exerted its effects by the activation of peroxisome proliferator-activated receptor alpha (PPARa), which is a transcription factor involved in regulating fatty acid catabolism and utilization. It was concluded that* C. pinnatifida* extract improved dyslipidemia and decreased body weight, so it can potentially be useful in the treatment of obesity and dyslipidemia (44).

Overall *C. pinnatifida *demonstrated the ability to reduce the expression of lipogenic genes, reduce lipid droplet formation and TG accumulation, decrease the expression of adipogenic transcription genes (PPARg and C/EBPa mRNA) and promote lipolysis by stimulating glycerol release. It also displayed abilities to control weight gain and serum lipid levels, by activating peroxisome proliferator-activated receptor alpha (PPARa), inhibiting adipose tissue accumulation and increasing excretion of lipids through feces. However, more experiments should be considered in order to identify the exact active components responsible for the therapeutic effects observed.

**Anti–hyperlipidemia effect**

High cholesterol levels initiate dyslipidemia and diseases such as fatty liver disease. By reducing serum cholesterol levels, we can control the onset of cardiovascular diseases (45). Dyslipidemia which consists of hypercholesterolemia, hypertriglyceridemia or the combination of both is considered an important factor in the development of atherosclerosis (46).

Pectin is a soluble fiber made up of acid polysaccharides with a galacturonic backbone (47). It has several applications in the industry for example it is used as a stabilizer, thickener or gelling agent in the food industry (48). It also has favorable biological effects in the body, like encouraging the growth of intestinal probiotics (49) or removing heavy metals which are toxic from the body and lowering fat by acting as a soluble fiber (48). In a 2013 study, the antioxidant and anti-lipogenic effects of haw pectin penta-oligogalacturonide (HPPS) on the liver of mice fed with a high fat diet were tested. The hawthorn fruit was prepared through several steps, drying, slicing, hot water extraction, hydrolyzing with pectinase and several other steps (the complete protocol can be found in the main article). The animals used were six week old male Kunming mice that weighed 20-25 g (pathogen free). Ten weeks after the start of the experiment results showed that hepatic TG levels in the normal control and high fat fed diet group were, 17.4±2.81 and 45.6±3.65 mg/g, respectively. The HPPS treated group had hepatic TG levels of 37.32±3.31, 29.72±3.44 and 27.3±5.38 mg/g for doses of 50, 150, and 300 mg/kg, respectively. It showed that the dose 300 mg/kg was able to suppress hepatic TG by as much as 40%. At higher doses (150-300 mg/kg), HPPS treatment was also able to suppress epididymal and perirenal fat accumulation, indicating that HPPS had the ability to block adipose tissue accumulation in mice fed a high fat diet. Results of the liver histology also showed that HPSS administration was able to reduce lipid inclusions in the liver by number and size as well. To conclude, haw pectin could be used as a liver protector and to stop hyperlipidemia, and the diseases that follow it such as, fatty liver and cirrhosis. The results of enzyme analysis studies suggested the theory that, the serum TG lowering effects of *C. pinnatifida *are due to its ability to increase hepatic FA oxidation enzyme activity. Results also showed an increase in PPARa expression, suggesting that HPSS might activate PPARa and as a result, up-regulating the expression of hepatic FA oxidation genes (50).

In a previous study, Zang *et al*. 2002 discovered that the aqueous ethanolic extract of *C. pinnatifida* could inhibit the acyl-coA:cholesterol acyltransferase (ACAT) enzyme in the intestine of hamsters. However, the specific compounds that could be responsible for this function were not discovered, this was the aim of a recent study. In order to further investigate, extracts were prepared from hawthorn fruits. The extracts were first tested on Caco-2 cells (*in vitro*). Results showed that extracts containing higher concentrations of oleanolic acid (OA) and ursolic acid (UA) (two triterpenic acids) showed ACAT inhibitory effects. Then OA and UA were purified and used in the hamster study. Male Lakeview golden Syrian hamsters were fed a specific diet (semisynthetic diet) containing cholesterol (Control) or the same diet with OA/UA extracts. Results exhibited that OA and UA reduced LDL and very low-density lipoprotein (VLDL) cholesterol levels by 6% compared to the control. In conclusion OA and UA reduced cholesterol levels by inhibiting the ACAT enzyme (51).

In a 2010 study 3-hydroxy-3-methylglutaryl coenzyme

A reductase (HMGR) inhibitors were isolated from hawthorn fruit and tested for anti-hyperlipidemic effects. HMGR is an enzyme that binds to the endoplasmic reticulum and catalysis the transformation of hydroxyl methyl glutaryl-CoA to mevalonate (52). By doing this HMGR stops the formation of cholesterol early on and thus reduces cholesterol levels. In order to study the effects of hawthorn, an ethanol extract was prepared from the fruit and then the extracts were purified with silica and column chromatography. The fractions with high HMGR inhibitory effects were obtained and further studied. Results identified four main HMGR inhibitor compounds, quercetin (Figure 2A), hyperoside (Figure 2B), rutin (Figure 2C) and chlorogenic acid (Figure 2D). Both *in vivo* and *in vitro* studies showed that the compounds had synergetic effects and maximum inhibitory effects were seen when a mixture of the compounds were used. Structure activity relationship studies showed that lipid lowering and HMGR inhibitory effects were related to the number of glycosyl groups in the compounds. The compounds with the most glycosyl groups had the least lipid lowering effects. Quercetin which had no glycosyl group showed the most effect (53). The chemical structures of the different compounds, which were extracted from the hawthorn fruit, are shown in Figure 2.

In 2014, Hu *et al. *performed a study in which the effects of a multiherbal preparation containing *C. pinnatifida* and several other herbs including *Alisma orientalis, Stigma maydis, Ganoderma lucidum, Polygonum multiflorum *and* Morus alba* on dyslipidemia were examined. Participants in the study were 42 Chinese patients 18 years or older that were considered to have dyslipidemia. The participants had to take four capsules in the morning and in the evening for a period of 12 weeks. Patients were checked at the sixth and twelfth week as well as at the baseline of the experiment. Lipid profiles were measured using biochemistry measurements. Results showed a 9.4% decrease of LDL-C levels in patients who had received the herbal formula compared to those who received the placebo. Regarding the small sample size of this study, the study was unable to detect small differences between the groups. The research also exhibited that the herbal formula elevated TG levels after 12 weeks. In the end it was concluded that the multiherbal formulation showed only marginal beneficial effects on LDL-C levels. The study did not determine specific active compounds. Adverse effects such as stomach upset, cough, influenza, shoulder pain, knee pain and headaches were noticed but not declared clinically significant (54).

Another multiherbal preparation containing *Aurricularia aurricula, Pueraria radix *and *C. pinnatifida *(AHP) was tested on 4-week-old ICR mice that were fed a cholesterol-enriched diet (CED) for 12 weeks. Blood samples showed a significant decrease in LDL-C levels. At the dose of 150 mg/kg/day, AHP reduced LDL-C by 22.92 % and at the dose of 450 mg/kg/day LDL-C decreased by 39.58%. Total cholesterol levels were also reduced to 75.13% (150 mg/kg/day) and 68.81 % (450 mg/kg/day) of the initial levels in the CED group. AHP also showed the ability to decrease fatty deposits in the liver of the treated mice, thus showing potential in the treatment of fatty liver disease (55).

Clinical studies have shown that post-menopausal women and ovariectomized patients are at a higher risk for cardiovascular diseases due to estrogen-deficiency–induced oxidative stress (56, 57). Higher levels of total cholesterol, LDL-C and VLDL levels have been observed (58) and studies have shown that post-menopausal women are at risk for metabolic disorders like hypertension, dyslipidemia, vascular inflammation, type 2 diabetes and endothelial dysfunction (59). In a study, the effects of *C. pinnatifida* fruit extracts on ovariectomized rat’s lipid profiles were studied as a mimic model for menopause. First, the extract was prepared from hawthorn fruit powder. The animals used were nine-week-old female Sprague–Dawley rats. A group of these rats were ovariectomized (OVX) and the others were not (Sham group), and then they were divided into 4 groups. Some groups becoming the controls and the others the OVX rats receiving either 100 mg/kg of the hawthorn extracts (OL) or 200 mg/kg of the extract (OH). After that, the serum lipid profiles were analyzed. The results showed that total cholesterol levels were reduced by 16% in the OL group and 14% lower in the OH group compared to the OVX control group, which did not receive hawthorn fruit extract. Also LDL levels decreased by 20 and 32% in the OL and OH groups, respectively. Free fatty acids decreased by 51% in the OH group compared to the OVX control. However, no significant difference was observed in triglyceride and VLDL levels. This study shows that hawthorn fruit extract could potentially be useful in improving lipid profiles in post-menopausal women (57).

In a different study, the effects of Shan zha (fruit of *Crataegus pinnatifida*) on blood lipid levels in mice fed a high fat diet were evaluated. C57BL/6J male mice were first fed a high fat diet (HFD) for 12 weeks. Then, 250 mg/kg Shan zha (which was dissolved in water) was administered by gavage orally 3 times a day for a week. Blood samples from the mice were collected. According to the results, treatment with Shan zha was able to lower both TG and cholesterol levels. However, it did not reduce body weight in the HFD mice. The study also examined the effects of Shan zha on PPAR-α expression in white adipose tissue in mice with hyperlipidemia, but found that Shan zha had no effect in this area (60).

Previous studies have also suggested that Shan zha extract performed its cholesterol lowering effect by up regulating CYP7A1 mRNA expression in the liver. As a result, bile acid biosynthesis is increased with augmentation of cholesterol expenditure (61).

The effects of *C. pinnatifida *dried fruit on hypercholesterolemia in rats, fed a high cholesterol diet (HCD), was examined in a 2010 study. In this study Male Sprague –Dawley rats were randomly fed one of three possible diets of, normal control diet, HCD diet or HCD plus hawthorn dried fruit powder. After a 4-week period, plasma lipid profiles showed the group that received the hawthorn fruit powder, had reduced total cholesterol (HCD-fed, 6.06±0.19mM; hawthorn fruit powder plus HCD-fed, 4±0.48) and LDL-lipoprotein levels. They also showed an increased HDL-lipoprotein level as well. Overall the results showed that consumption of Hawthorn fruit powder for 4 weeks was able to control and reduce plasma lipids in HCD fed rats (62).

In order to investigate the blood lipid lowering effects of *C. pinnatifida *in rats and humans, a study was performed by creating a low sugar content drink from hawthorn. It was also fortified with vitamin c and zinc gluconate to enhance the anti-oxidative properties of the plant. In the animal study, 37 male Sprague-Dawley rats were divided into three groups and either fed the hawthorn drink, 8% sugar water (control group) or tap water (secend control group). Results showed that after 6 to 9 weeks of treatment with the hawthorn drink, the body weight of the rats receiving the treatment was lower than the other groups, but was non-significant. Interestingly after the tenth week of treatment, the group receiving the hawthorn drink showed significantly lower body weights. The serum cholesterol and TG levels were also reduced significantly. Results also showed that the hawthorn drink was able to increase HDL-C levels and decrease LDL-C levels the rats. In the human study 30 subjects that were diagnosed with hyperlipidemia, took the hawthorn drink twice a day for a month, they also stopped taking their other medications. The patients had an initial average serum cholesterol level of 7.30 ±1.04 mmol/l which was reduced to 6.19±1.56 mm0l/l after one month of drinking the hawthorn drink. Their TG levels also decreased from 1.93±0.92 to 1.75±0.96 mmol/l. The drink was also able to reduce LDL-C and apo-B levels. The hawthorn drink also showed strong anti-oxidative effects due to its ability to reduce lipid peroxidate malonic dialdehyde levels (63).

To sum up, *C. pinnatifida *showed anti–hyperlipidemia potential through various mechanisms such as increased hepatic FA oxidation enzyme activity, ACAT enzyme inhibition, HMGR enzyme inhibition, increased bile acid biosynthesis, strong anti-oxidative properties and reduction of apo-B and lipid peroxidate malonic dialdehyde levels. More studies should be done in order to explore the specific mechanisms of active compounds.

Different studies on anti–hyperlipidemia effects of *C. pinnatifida *have been summarized in Table 1.

**Effects on atherosclerosis**

Atherosclerosis (AS) is a disease that highly involves in mortality all around the world (64). AS can cause heart diseases such as acute coronary syndrome and strokes. Data has shown that there is a strong relationship between the amount of lipids in the plasma and cardiovascular problems (65).

In a study, the effects of an aqueous extract of *Crataegus pinnatifida* fruit (AECP) on serum lipid levels were tested. The animals used in the experiment were 40 male Wistar rats. The rats were randomly divided groups. Normal model, model plus simvastatin (10 mg/kg), model plus low dose AECP (L-AECP 72 mg/kg) and model plus high dose AECP (H-AECP 288 mg/kg). Beginning from the ninth week, simvastatin and the low and high dose AECP were administered via the intragastric route every day for four weeks. Total cholesterol, LDL-C, TG and HDL-C levels were measured using a biochemistry analyzer. Results showed that L-AECP was able to decrease total cholesterol and H-AECP was able to reduce total cholesterol, TG and LDL-C levels, while both L-AECP and H-AECP were able to increase HDL-C levels. Additionally, AECP significantly reduced the expression of some important cytokines. In conclusion AECP has useful effects in reducing and preventing atherosclerosis (66).

Recently Dong *et al.* studied the effects of hawthorn leaf flavonoids (HLF) on the development of atherosclerosis in apoE knock-out mice. Male apoE knock-out mice were either fed HLF (5 or 20 mg/kg/day) or normal chow for 16 weeks, then the mouse aortas were dissected and the face aortic lesion area was observed. Results exhibited that the atherosclerotic lesion area in the mice fed HLF was significantly reduced (23.1%) compared to the control group (67).

The seeds of *C. pinnatifida* also have therapeutic properties*. *Six new compounds from the seeds of *C. pinnatifida* were collected and investigated for their potential antithrombotic effects. To determine the antithrombotic activities of these six compounds the platelet aggregation method was used (adenosine diphosphate induced), and aspirin was used as the positive control. Among the six compounds, one compound, a sesquiterpene (1-isoprpranol-4a-methylendecahydronaphthalene) was able to inhibit platelet aggregation in rat plasma by 81.42% at 1 mg/ml concentration. Thus, (1a, 4ab, 8aa)-1-isopropanol-4a-methyl-8-methylenedecahydronaphthalene showed *in vitro* antithrombotic activity (the structure of this compound can be seen in Figure 3A) (68).

In 2015, new compounds from the leaf of *C. pinnatifida* were isolated and structurally characterized. These compounds were tested for *in vitro* and *in vivo* antithrombotic effects. The *in* *vitro* study was carried out with the platelet aggregation method, which was induced by ADP and aspirin was used as the positive control. Three compounds, (6S, 7Z, 9R)–roseoside (Figure 3B), eriodectyol (Figure 3C) and 2”-O- rhamnosyl vitexin (Figure 3D) displayed antithrombotic activity at a dose of 400 mg/ml, by inhibiting platelet aggregation in rat plasma by 87.17, 72.92, and 75 % compared to aspirin which inhibited platelet aggregation by 92.20% at the same concentration. We can see the structure of the three compounds in Figure 3. In the *in vivo* study, the time to form a thrombus induced by ferric chloride was tested in Zebrafish. Results showed that the three compounds mentioned previously, inhibited thrombus production. Among these three compounds, eriodectyol (19.04±3.32 min) was able to significantly prolong the time it took to form a thrombus compared to the control group (17.63±2.23 min) (69). Figure 3 shows the structure of the compounds that showed antithrombotic activity.

## Conclusion

From the data collected, we can conclude that *C. pinnatifida *has a vast array of effects on factors contributing to the formation of metabolic syndrome. It can be beneficial in diabetes, obesity, hyperlipidemia and atherosclerosis. Active components in leaves, fruits and seeds are responsible for its pharmacological effects. The inhibition of ACAT and HMGR enzymes, reduction of platelet aggregation and reducing lipid inclusions in the liver have been noticed as main mechanisms that are involved in beneficial properties of C*. pinnatifida. *With further clinical and safety studies, *C. pinnatifida *could show the potential of becoming a new and safe medicine in the market for the treatment of metabolic syndrome.

## Conflicts of Interest

The authors declare there is no conflict of interest.
